# A positive feedback loop of kynurenine-AhR-EMT upregulates tryptophan metabolic enzymes via epigenetic modification to facilitate immune escape in triple-negative breast cancer

**DOI:** 10.1080/23723556.2026.2712774

**Published:** 2026-10-14

**Authors:** Chen Jun, Li Menghui, Liu Ting, Chen Juan

**Affiliations:** a Department of Breast Surgery, Nanchang People's Hospital, Nanchang, China

**Keywords:** Triple-negative breast cancer, kynurenine, aryl hydrocarbon receptor, epithelial-mesenchymal transition, immune escape

## Abstract

**Background:**

Triple-negative breast cancer (TNBC) is prone to immune escape, and the synergistic mechanism of kynurenine, AhR, and EMT remains unclear.

**Methods:**

TNBC cell lines and 4T1 orthotopic breast cancer mice were used for molecular, cellular, and in vivo intervention experiments.

**Results:**

Kynurenine induced EMT in an AhR-dependent way, promoted cell migration and invasion, upregulated CCL2/CCL22, and inhibited the function of CD8⁺GrB⁺ T cells. Snail overexpression promotes H3K4me3 histone modification at IDO1 and TDO2 promoters to epigenetically amplify tryptophan metabolic enzyme expression. The AhR antagonist CH-223191 blocked the loop and reversed tumor immune escape.

**Conclusion:**

This study demonstrates that the kynurenine-AhR-EMT positive feedback loop drives immune escape in TNBC, providing a rationale for combined targeted therapy.

## 1. Introduction

Triple-negative breast cancer (TNBC) is the most aggressive subtype of breast cancer, characterized by the absence of estrogen receptor, progesterone receptor, and human epidermal growth factor receptor 2, which renders targeted therapies against these molecules ineffective.^1^ The high heterogeneity and invasive phenotype of TNBC are closely associated with the complex immunosuppressive network in the tumor microenvironment. Typically, the microenvironment presents reduced infiltration of CD8⁺ T cells and enrichment of immunosuppressive myeloid cells and regulatory T cells, forming a “cold tumor” state.^2^ ^,^ ^3^ Although immune checkpoint inhibitors have achieved breakthroughs in the clinical treatment of TNBC, most patients develop resistance due to intrinsic or acquired tumor immune escape mechanisms, leading to unsatisfactory overall response rates.^2^ ^,^ ^4^

In recent years, numerous studies have confirmed that multiple molecular mechanisms participate in TNBC immune escape, including metabolic reprogramming, abnormal epigenetic modification, and dysregulated expression of immune checkpoint molecules.^5^ ^,^ ^6^ For instance, excessive activation of the MYC signaling pathway,^4^ aberrant expression of MUC1-C,^7^ and activation of the integrin αvβ6-TGFβ-SOX4 pathway^8^ can suppress tumor immune surveillance through distinct mechanisms. Despite these findings revealing the diversity of TNBC immune escape, the mechanism by which tryptophan metabolites directly drive tumor cell phenotypic remodeling and construct an immunosuppressive microenvironment remains poorly understood, which requires further exploration to identify novel therapeutic targets.

As a ligand-activated transcription factor, aryl hydrocarbon receptor (AhR) plays a vital role in the initiation and progression of various tumors. Previous studies have shown that AhR is abnormally overexpressed in TNBC. Its activation directly drives malignant transformation, including promoting cell proliferation, enhancing migration and invasion, and inducing epithelial-mesenchymal transition (EMT).^9^ Environmental pollutants such as bis(2-ethylhexyl) phthalate (DEHP) can also activate the AhR signaling pathway, upregulate TFF3 expression and induce EMT, thereby facilitating drug resistance and recurrence of breast cancer.^10^

EMT is a key biological process for tumor cells to acquire invasive and metastatic capabilities, which is precisely regulated by core EMT transcription factors (EMT-TFs) including SNAI1, SNAI2, ZEB1, ZEB2 and TWIST1.^11^ A large number of clinical studies have verified that EMT-TFs are abnormally upregulated in multiple solid tumors, and their high expression is significantly correlated with poor patient prognosis. For example, overexpression of SNAI1 and ZEB1 indicates shorter overall survival and advanced clinical stages in non-small cell lung cancer, pancreatic ductal adenocarcinoma, and head and neck squamous cell carcinoma.^12^ ^,^ ^13^

Kynurenine (Kyn), a pivotal metabolite of tryptophan, is generated via reactions catalyzed by indoleamine 2,3-dioxygenase 1 (IDO1) and tryptophan 2,3-dioxygenase 2 (TDO2) in the tumor microenvironment. It has been identified as a major endogenous ligand of AhR. Dysregulated tryptophan metabolism in the tumor microenvironment has been shown to be closely linked to malignant progression and immune escape of breast cancer. Tryptophan is metabolized via the kynurenine pathway to produce Kyn, which acts as a critical endogenous ligand to activate the AhR signaling pathway.^14^ Notably, the loss of miR-200c expression in TNBC leads to the upregulation of its target gene TDO2, which increases kynurenine production and maintains the EMT phenotype along with elevated expression of immunosuppressive factors.^15^

Although disparate lines of evidence have indicated potential correlations among the Kyn-AhR axis, EMT, and the expression of tryptophan metabolic enzymes, it remains unclear whether these three factors form a synergistic positive feedback loop to amplify the immunosuppressive microenvironment of breast cancer. Tumor progression is frequently modulated by interconnected signaling axes with multidimensional regulatory characteristics in solid cancers.^16^ Moreover, molecules regulating the tumor immune microenvironment are widely recognized as valuable prognostic markers and therapeutic candidates for solid tumors.^17^ Therefore, this study aimed to explore whether the Kyn-AhR axis drives EMT and further feedback to upregulate tryptophan metabolic enzymes (IDO1/TDO2), forming an autocrine or paracrine positive feedback loop that promotes the immunosuppressive microenvironment of TNBC. By combining in vitro cellular experiments, co-immunoprecipitation, ChIP-qPCR, and in vivo 4T1 orthotopic breast cancer mouse model with AhR antagonist intervention, we systematically verified the critical role of the Kyn-AhR-EMT positive feedback loop in breast cancer immune escape, thereby providing novel combined immunotherapeutic strategies targeting this tryptophan metabolism-EMT loop.

## 2. Materials and methods

### 2.1. Cell lines and cell culture

Human TNBC cell lines MDA-MB-231, BT549, mouse TNBC cell line 4T1, and human embryonic kidney cell line HEK293T were purchased from the American Type Culture Collection (ATCC). MDA-MB-231, BT549, and HEK293T cells were routinely cultured in high-glucose DMEM medium (HyClone) supplemented with 10% fetal bovine serum (FBS, Gibco). 4T1 cells were cultured in RPMI-1640 medium (HyClone) containing 10% FBS. All culture media were supplemented with 100  U/mL penicillin and 100 μg/mL streptomycin. Cells were incubated in a humidified incubator at 37 °C with 5% CO₂ and passaged every 2 to 3 d.

### 2.2. Reagents and antibodies

L-kynurenine (Kyn, Cat. No.K8625) was purchased from Sigma-Aldrich. The AhR antagonist CH-223191 (Cat. No.HY-12684) was obtained from MedChemExpress. siRNA/shRNA targeting sequences for AhR and Snail were synthesized by Shanghai GenePharma Co., Ltd. The primary antibodies used included rabbit anti-human AhR(Cat. No.ab314060, Abcam), Rabbit anti-human Snail (Cat. No.3879, SCT), mouse anti-human/mouse CD8 (Cat. No.12-0081-83, eBioscience), and Granzyme B (GrB, Cat. No.515403, BioLegend). anti-H3K4me3 antibody (Cat. No.9751S, CST), anti-GAPDH (Cat. No.ab8245, Abcam). All antibodies were diluted in accordance with the manufacturer's instructions.

### 2.3. Plasmid construction and cell transfection

The Snail overexpression vector was cloned into the pcDNA3.1 plasmid. Transient transfection was performed using Lipofectamine^TM^ 3000 (Cat. No. L3000008, Invitrogen). Stable cell lines were established via lentiviral transduction using particles carrying target genes or shRNA (packaged by Shanghai Hanheng Biotechnology Co., Ltd.), followed by screening with 2 μg/mL puromycin. For siRNA transfection, Lipofectamine^TM^ RNAiMAX (Cat. No. 13778150, Invitrogen) was applied. Follow-up experiments were conducted 48 to 72 hours after transfection.

### 2.4. Co-immunoprecipitation (Co-IP) and Western blot

Cell lysates were prepared using RIPA buffer supplemented with protease inhibitors (Cat. No. 04693132001, Roche). Equal amounts of protein (1–2 mg) were incubated with corresponding antibodies (2 μg) overnight at 4 °C. Subsequently, Protein A/G agarose beads (Cat. No. sc-2003, Santa Cruz) were added and incubated for 2 hours with rotation. Eluted protein samples were denatured by boiling. For Western Blot assay, proteins were separated by SDS-PAGE and transferred onto PVDF membranes (Millipore). Membranes were blocked with 5% skim milk powder, incubated with primary antibodies overnight at 4 °C, and then incubated with HRP-conjugated anti-GAPDH secondary antibodies (Cat. No.ab8245, Abcam) at room temperature for 1 hour. Protein bands were visualized using ECL chemiluminescence reagent (Cat. No.32209, Pierce).

### 2.5. Chromatin immunoprecipitation-quantitative PCR (ChIP-qPCR)

ChIP experiments were performed using the SimpleChIP Plus Enzymatic Chromatin IP Kit (Cat. No. 9005S, CST). Cells were cross-linked with 1% formaldehyde for 10 minutes, and glycine was added to terminate cross-linking. Chromatin was fragmented into 200–500 bp segments by ultrasonication. Equal amounts of chromatin were incubated with H3K4me3 antibody or IgG (negative control) overnight at 4 °C, and enriched using Protein G magnetic beads. After elution and reverse cross-linking, DNA was purified. qPCR was used to detect the enrichment level of H3K4me3 in the promoter regions of IDO1 and TDO2. Data were presented as percentage of input (% input).

### 2.6. Immunofluorescence (IF)

Cells were fixed with 4% paraformaldehyde, permeabilized with 0.1% Triton X-100, and blocked with 5% BSA. Cells were incubated with primary antibodies overnight at 4 °C, followed by incubation with Alexa Fluor 488/594-conjugated fluorescent secondary antibodies (Cat. No.A11008/A11005, Invitrogen) for 1 hour at room temperature in the dark. Nuclei were counterstained with DAPI. AhR nuclear translocation was observed under a Zeiss LSM 880 confocal microscope. For each group, at least five random fields from three independent experiments were acquired for representative images and quantitative analysis of corrected mean fluorescence intensity (MFI).

### 2.7. Flow cytometry

For in vitro T-cell functional detection, flow cytometry was performed on co-cultured human T lymphocytes. Briefly, treated T cells were fixed, permeabilized, and stained with fluorescently labeled antibodies (CD8-FITC, GrB-PerCP) for 30 minutes at 4 °C in the dark. Samples were acquired using a BD FACSCanto II flow cytometer, and data were analyzed with FlowJo software to quantify the proportion of tumor-infiltrating CD8⁺GrB⁺ cells.

### 2.8. Enzyme-linked immunosorbent assay (ELISA)

Supernatants of cell culture medium or tumor tissue homogenate were collected. Commercial ELISA kits were used to detect the concentrations of CCL2 (JL19334, JONLN BI0, Shanghai, China) and CCL22 (SP10226, Saipai Bio, Wuhan, China). Human TDO ELISA kit (JL45807, JONLN BI0, Shanghai, China), human IDO1 ELISA kit (ml037385, Meilian Bio, Shanghai, China), and kynurenine ELISA kit (ml229877, Meilian Bio, Shanghai, China) were also used in accordance with manufacturer protocols. The absorbance at 450 nm was measured using a Bio-Rad iMark microplate reader, and sample concentrations were calculated based on standard curves.

### 2.9. Cell migration and invasion assays

24-well Transwell chambers (8 μm pore size, Corning) were used for migration and invasion assays. For the migration assay, 2 × 10^4^ cells were seeded in the upper chamber with serum-free medium, and medium containing 10% FBS or conditioned medium was added to the lower chamber. For the invasion assay, the upper chamber was pre-coated with diluted Matrigel (1:8, BD Biosciences), and 5 × 10^4^ cells were seeded. Following incubation for 24 hours (migration) or 48 hours (invasion), non-migrated cells on the upper membrane surface were gently removed. Cells were fixed with 4% paraformaldehyde and stained with 0.1% crystal violet. Cells in five random fields were counted for statistical analysis.

### 2.10. Cell Co-culture and T-cell differentiation analysis

Peripheral blood mononuclear cells (PBMCs) were isolated from peripheral blood samples of healthy volunteers using lymphocyte separation medium. PBMCs were seeded in culture flasks pre-coated with anti-human CD3 antibody and recombinant human fibronectin. IL-2 and IFNγ were added to 1640 medium to expand CD3⁺ T lymphocytes. After 5 d of culture, 1 × 10^5^ T lymphocytes per well were co-cultured with tumor-conditioned medium collected from kynurenine-treated groups for 24 hours. PMA (100 μg/L) and ionomycin (1 mg/L) were added at 19 and 43 hours of incubation, respectively. T lymphocytes cultured alone under the same conditions were set as the control group. After another 5 hours of incubation, T cells were collected for flow cytometry detection.

T cells were fixed with 4% paraformaldehyde for 5 minutes and permeabilized with 0.3% Triton X-100 for 5 minutes. FITC-conjugated CD8 antibody and Granzyme B antibody diluted in 200 μL PBS were added and incubated on ice for 30 minutes. After centrifugation and washing three times with PBS, the samples were analyzed by flow cytometry.

### 2.11. Animal experiments

Female BALB/c mice (6–8 weeks old; body weight 18–20 g) were purchased from Beijing Vital River Laboratory Animal Technology Co., Ltd. and housed in a specific pathogen-free (SPF) facility.. The 4T1 orthotopic TNBC model was established by injecting 1 × 10^5^ 4T1 cells into the fourth mammary fat pad of mice. Mice were randomly divided into six groups (8–10 mice per group): (1) Control group (normal saline); (2) Kynurenine group (100 mg/kg/d, intraperitoneal injection); (3) CH-223191 group (10 mg/kg/d, intraperitoneal injection); (4) Empty vector group; (5) Snail overexpression group; (6) Snail overexpression + CH-223191 group (10 mg/kg/d, intraperitoneal injection). The long and short diameters of tumors were measured every 7 d, and tumor volume was calculated by the formula: *V = length × width^2^ × 0.5*. At the end of the experiment, tumor tissues were harvested for subsequent detection of AhR, Snail, IDO1/TDO2 expression, and immune cell infiltration.

### 2.12. Statistical analysis

All data are presented as mean ± standard deviation (mean ± SD) from at least three independent experiments. Two-group comparisons were performed using two-tailed Student's t-test. Multiple-group comparisons were analyzed by one-way analysis of variance (ANOVA) followed by Tukey's multiple comparison test. All statistical analyzes were conducted using GraphPad Prism 9.0. A *p*-value less than 0.05 was considered statistically significant.

## 3. Results

### 3.1. Kyn activates AhR and drives EMT in TNBC cells

To explore the effect of kynurenine on EMT in TNBC cells, MDA-MB-231 and BT549 cells (TNBC cell lines with active AhR pathway, strong EMT phenotype and high metastatic potential) were treated with kyn. Cell proliferation assays showed that 5 μM kynurenine significantly inhibited the proliferation of MDA-MB-231 and BT549 cells (Figure 1A), so 1 μM kyn was selected for subsequent experiments. Immunofluorescence results revealed that AhR translocated from the cytoplasm to the nucleus 15 minutes after kyn treatment (Figure 1B).

**Figure 1.**
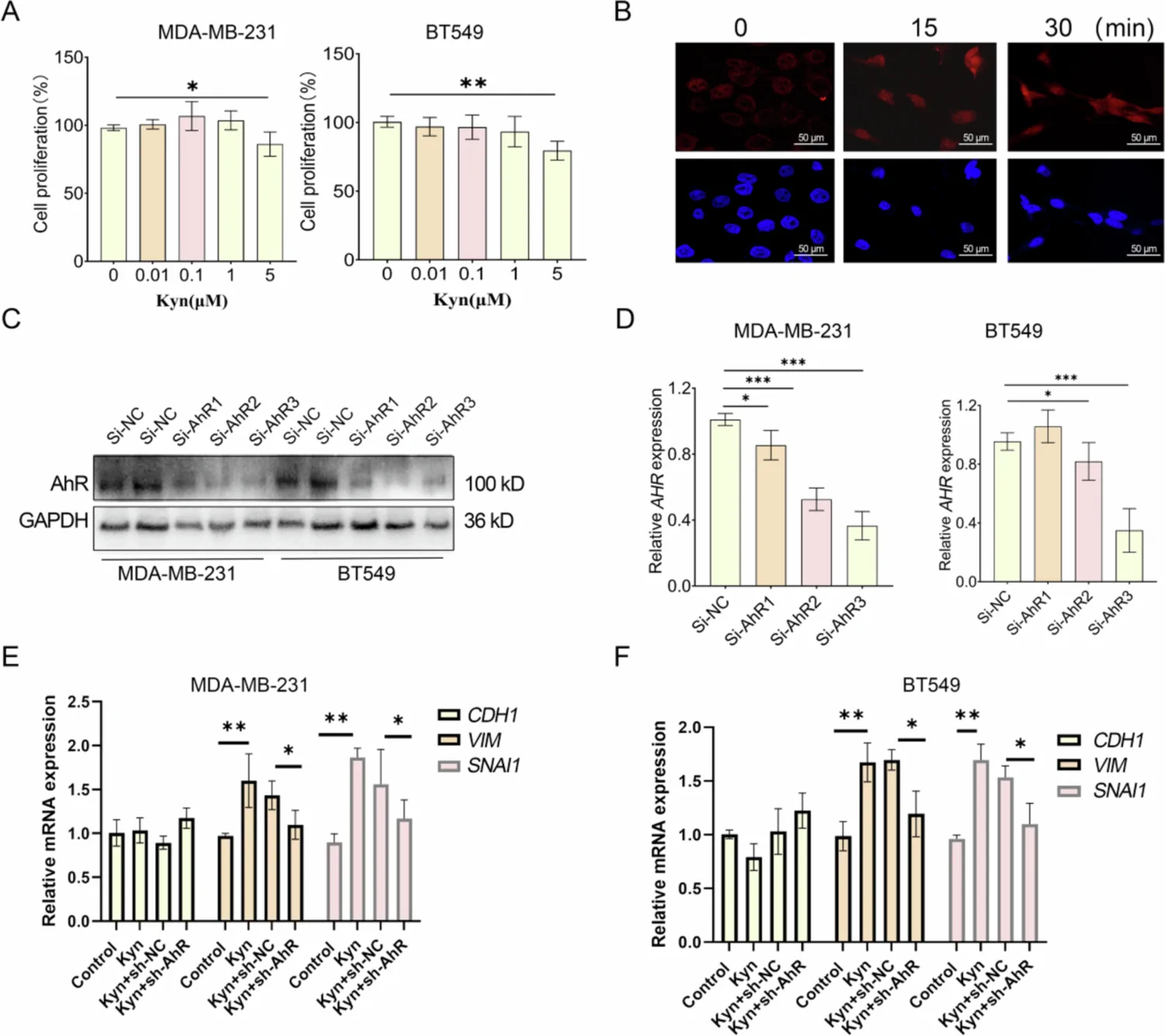
Kynurenine induces EMT in TNBC cells by activating AhR (*n* = 6 biological replicates). A: Proliferation curves of MDA-MB-231 and BT549 cells treated with serial concentrations of kynurenine (0, 0.01, 0.1, 1, 5 μM). B: Immunofluorescence staining showing time-dependent AhR nuclear translocation (0, 15, 30 minutes) after 1 μM kynurenine stimulation. C−D: Western blot and RT-qPCR analysis verifying AhR knockdown efficiency of three independent sh-AhR sequences. E−F: RT-qPCR quantification of mRNA levels of epithelial and mesenchymal signature genes, including *CDH1* (encoding E-cadherin), *VIM* (encoding Vimentin), and *SNAI1* (encoding Snail). ***p < 0.001, **p < 0.01, *p < 0.05.

Stable AhR knockdown cell lines were constructed, and sh-AhR-3 was verified to exert the highest knockdown efficiency (Figure 1C and D) and was applied for follow-up research. RT-qPCR results demonstrated that kyn treatment significantly upregulated the expression of mesenchymal marker Vimentin and EMT transcription factor Snail, while this upregulation was completely blocked by AhR silencing (Figure 1E and F).

### 3.2. AhR activation upregulates CCL2/CCL22 and recruits immunosuppressive cells

Transwell assays showed that 1 μM kyn markedly enhanced the migration and invasion abilities of TNBC cells, and AhR knockdown fully abrogated these effects (Figure 2A and B), indicating that this biological process is strictly AhR-dependent. ELISA results confirmed that kyn treatment increased the secretion of CCL2 and CCL22 in MDA-MB-231 and BT549 cells, an effect that was reversed by AhR knockdown (Figure 2C and D​​​​​​). In vitro differentiation experiments indicated that tumor-conditioned medium from kyn-treated groups impaired the differentiation of human CD8⁺GrB⁺ cytotoxic T cells, and this immunosuppressive effect was also blocked by AhR silencing (Figure 2E and F).

**Figure 2.**
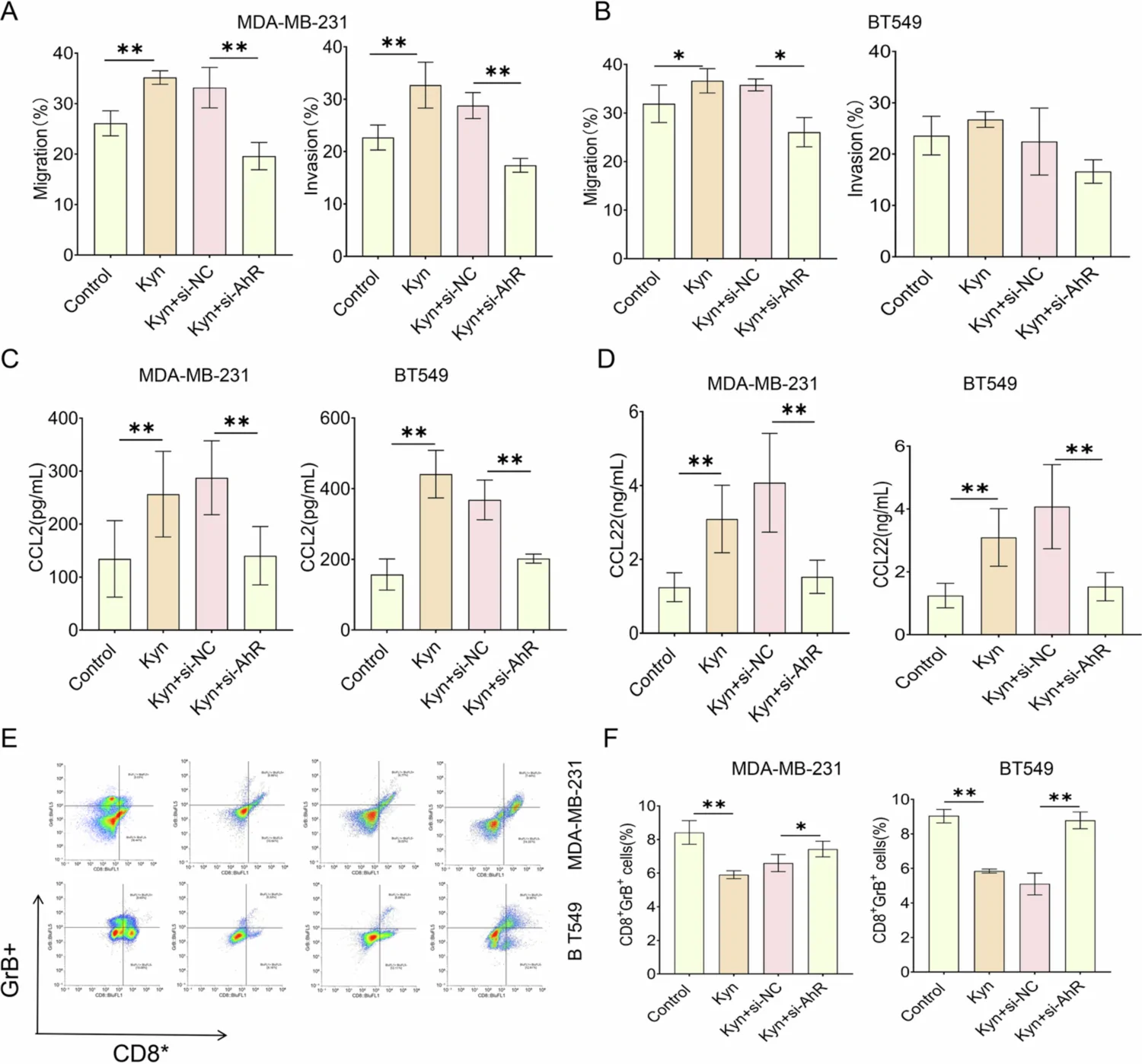
The Kyn-AhR axis promotes migration, invasion, and mediates immunosuppression in TNBC cells (*n* = 6 biological replicates). A−B: Transwell migration and invasion assays. For migration assays, 2 × 10^4^ cells were seeded in serum-free upper chambers; for invasion assays, chambers were pre-coated with diluted Matrigel (1:8), and 5 × 10^4^ cells were seeded. Migratory/invasive cells in five random fields were counted. C−D: ELISA detection of secreted CCL2 and CCL22 in cell culture supernatants from MDA-MB-231 and BT549 groups. E−F: Flow cytometry analysis of CD8⁺GrB⁺ cytotoxic T-cell proportion in an in vitro T-cell co-culture system. Tumor-conditioned medium from kynurenine-treated TNBC cells impaired CD8⁺GrB⁺T-cell differentiation, whereas AhR silencing reversed this immunosuppressive phenotype. Statistical markers: *p* < 0.01 and *p < 0.05.

### 3.3. EMT upregulates tryptophan metabolic enzymes via H3K4me3 epigenetic modification

We further explored whether EMT reversely regulated tryptophan metabolic enzymes to form a positive feedback loop. *SNAI1* was overexpressed in MDA-MB-231 cells (Figure 3A). The results showed that *SNAI1* overexpression increased the activity of IDO1 and TDO2, accompanied by elevated intracellular kyn production (Figure 3B). Conversely, *SNAI1* knockdown dramatically reduced the expression of IDO1, TDO2, and kynurenine levels (Figure 3C and D). ChIP-qPCR results showed that *SNAI1* overexpression significantly increased H3K4me3 enrichment in the promoter regions of IDO1 and TDO2, while AhR antagonist CH-223191 reversed this process (Figure 3E). *SNAI1* knockdown caused no obvious change in H3K4me3 enrichment (Figure 3F). To further verify whether Kyn-induced H3K4me3 deposition is AhR-dependent, we supplemented ChIP-qPCR experiments with an additional Kyn + CH-223191 co-treatment group. MDA-MB-231 cells were divided into four groups: Control (DMSO), 1 μM Kyn, 1 μM Kyn + 10 μM CH-223191, and single CH-223191 treatment. The results showed that Kyn treatment alone markedly elevated H3K4me3 enrichment at IDO1 and TDO2 promoters. Co-administration of the AhR antagonist CH-223191 completely abolished Kyn-triggered H3K4me3 accumulation, while CH-223191 alone exerted no significant effect on basal histone modification levels. These data confirm that Kyn-mediated epigenetic upregulation of IDO1/TDO2 fully relies on intact AhR signaling (Figure 3G). These findings demonstrate that Gain-of-function *SNAI1* overexpression significantly elevates H3K4me3 activation marks at IDO1/TDO2 promoters, while loss-of-function *SNAI1* silencing does not reduce basal H3K4me3 enrichment. These results indicate *SNAI1* acts as an epigenetic amplifier rather than an essential maintenance factor for the H3K4me3 modification of IDO1/TDO2 promoters.

**Figure 3.**
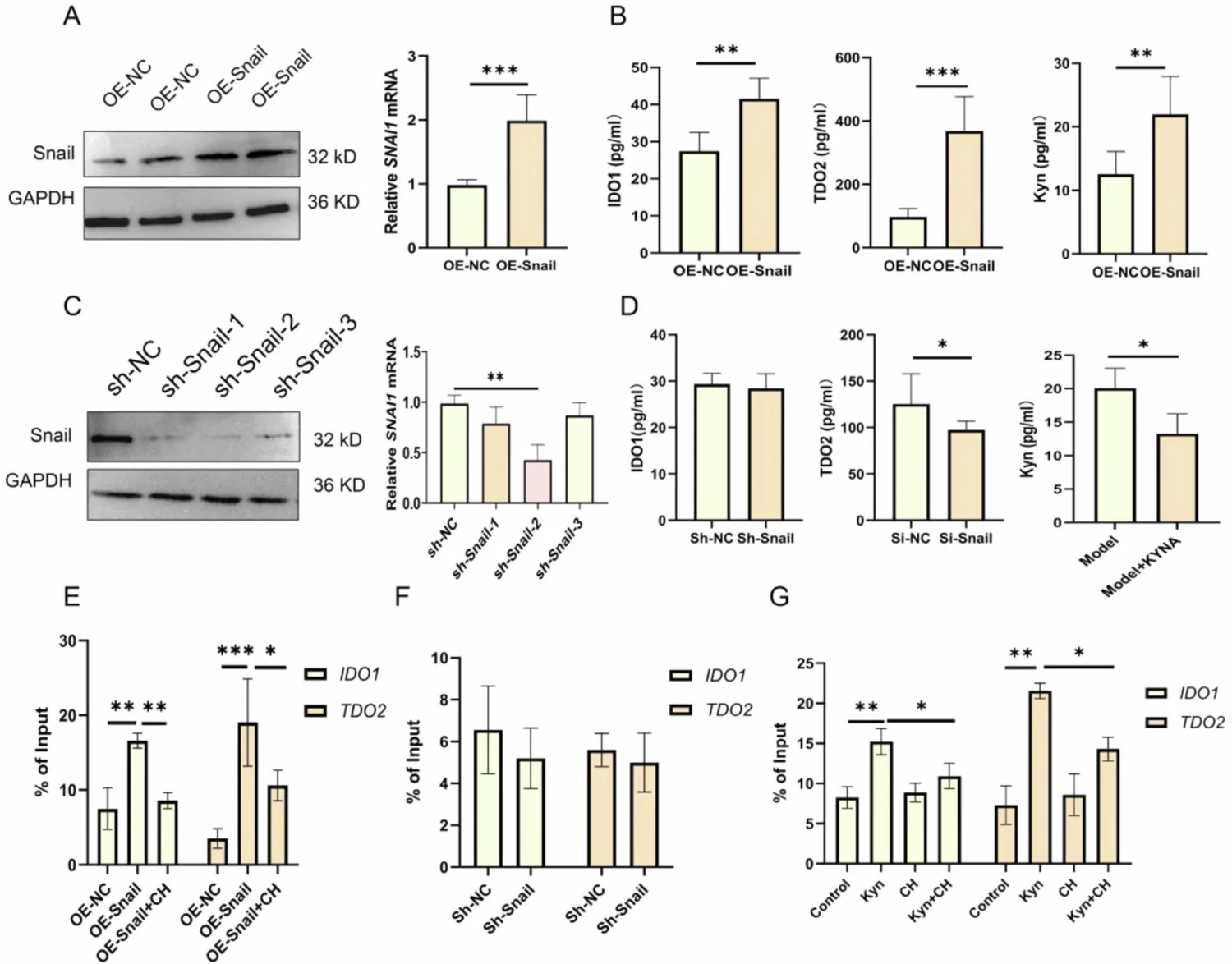
SNAI1 upregulates *IDO1* and *TDO2* expression via H3K4me3 epigenetic modification to boost intracellular kynurenine production (*n* = 6 biological replicates). A: Western blot and RT-qPCR validation of stable *SNAI1* overexpression efficiency in MDA-MB-231 cells. B: ELISA quantification of IDO1 and TDO2 protein and intracellular kynurenine concentrations upon *SNAI1* overexpression. C: Western blot and RT-qPCR validation of *SNAI1* shRNA knockdown efficiency. D: ELISA detection of IDO1, TDO2 and intracellular kynurenine after partial *SNAI1* knockdown. E–G: ChIP-qPCR analysis measuring H3K4me3 enrichment at the promoter regions of *IDO1* and *TDO2*, presented as percentage of input (% input). *SNAI1* overexpression significantly increased H3K4me3 enrichment, while partial shRNA-mediated SNAI1 knockdown produced no statistically significant changes. ***p < 0.001, *p* < 0.01, *p < 0.05.

### 3.4. The Kyn-AhR-EMT positive feedback loop is verified in vivo

The 4T1 orthotopic TNBC mouse model was used to verify the function of this positive feedback loop in vivo. The results showed that exogenous kyn significantly accelerated tumor growth, and the AhR antagonist CH-223191 reversed this phenotype (Figure 4A). Immunofluorescence detected increased AhR nuclear localization and CD8^+^ T-cell infiltration in tumor tissues (Figure 4B). We further quantified AhR and CD8α mean fluorescence intensity(MFI) across all in vivo groups (Figure 4C and D). RT-qPCR results revealed elevated expression of *Snai1* (Figure 4E). ELISA results revealed increased intratumoral expression of IDO1, TDO2, and kynurenine (Figure 4F), consistent with clinical LC-MS/MS metabolomics data reporting drastically elevated kyn concentrations in human TNBC tumor biopsies,^18^ as well as enhanced immunosuppression (increased Pdcd1, Cctla4, Lag3) in the kyn group (Figure 4G), all of which were rescued by CH-223191 intervention (Figure 4E−G).

**Figure 4.**
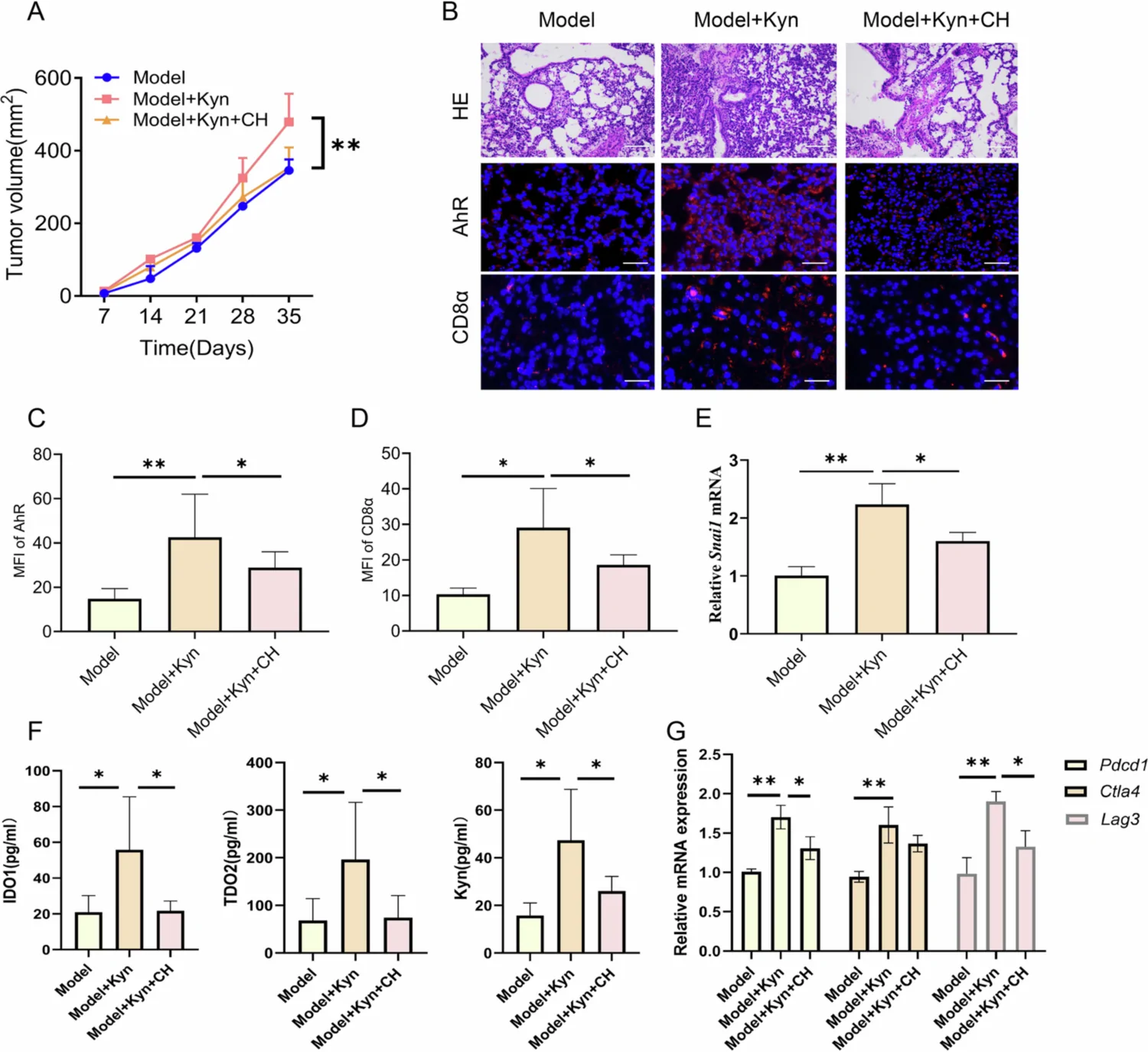
In vivo verification of the kynurenine-AhR signaling cascade regulating TNBC tumor growth and immune escape (*n* = 6–8 mice per group). A: Dynamic tumor volume growth curves of 4T1 orthotopic TNBC-bearing mice measured every 7 d; tumor volume was calculated as V = length × width^2^ × 0.5. B: Representative HE staining and AhR and CD8⁺ T-cell immunofluorescence images of tumor tissues, with 100 μm scale bars for HE sections and 50 μm scale bars. C,D: Quantitative analysis of AhR and CD8ɑ mean fluorescence intensity (MFI) in tumor IF fields. E: ELISA detection of IDO1, TDO2, and kynurenine levels in tumor homogenates. F: RT-qPCR measurement of *Snai1* mRNA expression in tumor tissues. G: RT-qPCR quantification of immune exhaustion markers *Pdcd1*, *Ctla4,* and *Lag3* mRNA in tumor tissues. CH, CH-223191. Statistical markers: **p < 0.01 and *p < 0.05.

Subsequent experiments on Snail overexpression showed that Snail overexpression also promoted tumor growth and aggravated immunosuppression. CH-223191 treatment effectively blocked these malignant phenotypes (Figure 5). The above results confirmed that Snail, the key EMT factor, maintains the activation of the Kyn-AhR-EMT positive feedback loop in vivo.

**Figure 5.**
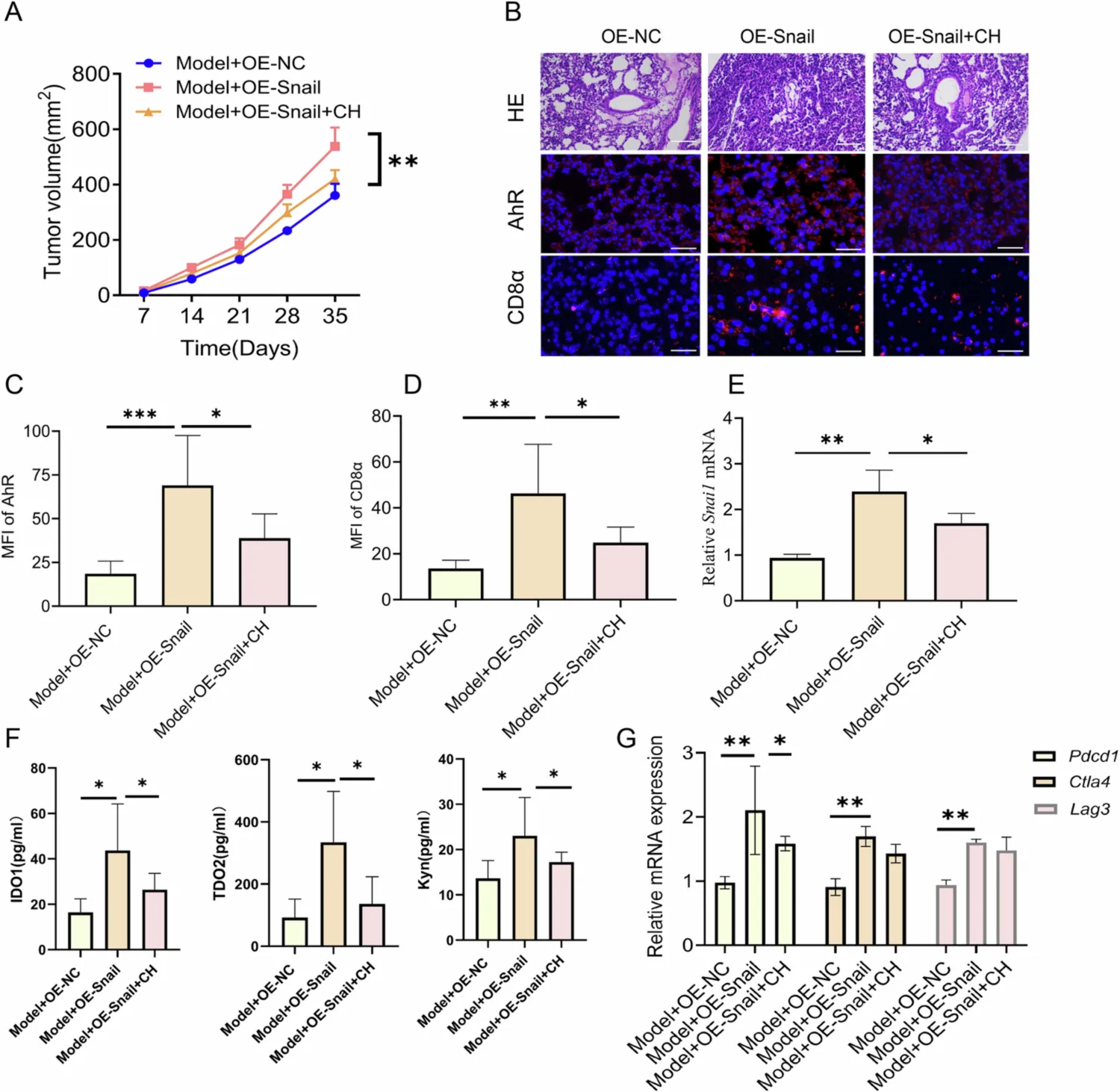
In vivo validation that SNAI1-mediated EMT sustains the Kyn-AhR positive feedback loop in TNBC (*n* = 6–8 mice per group). A: Tumor volume growth curves of mice injected with empty vector or SNAI1-overexpressing 4T1 cells, with or without CH-223191 intervention. B: Representative HE, AhR, and CD8^+^ IF images of tumor tissues, with 100 μm scale bars for HE sections and 50 μm scale bars for IF images. C,D: Quantitative analysis of AhR and CD8 ɑ MFI in tumor. E: ELISA detection of intratumoral IDO1, TDO2 and kynurenine concentrations. F: RT-qPCR detection of *Snai1* mRNA expression in tumor tissues. G: RT-qPCR quantification of exhaustion markers *Pdcd1*, *Ctla4* and *Lag3* mRNA levels. CH, CH-223191. Statistical markers: *p* < 0.01 and *p < 0.05.

## 4. Discussion

This study confirmed that tryptophan metabolite kyn activates AhR to induce EMT and upregulates chemokines CCL2/CCL22, thereby promoting the formation of immunosuppressive microenvironment in TNBC. Accumulating evidence indicates that AhR is frequently overexpressed in TNBC, and its abnormal activation is closely related to the malignant phenotype of tumor cells. As a major endogenous ligand of AhR, kyn is continuously produced by tumor cells via the tryptophan metabolic pathway to sustain persistent AhR activation.^19^ Exogenous AhR agonists such as environmental pollutants can also induce EMT to promote migration and invasion of breast cancer cells.^20^ The Kyn-AhR-EMT regulatory axis identified in this study provides a new insight into how metabolic microenvironment remodels tumor cell behaviors.

Activated AhR can bind to dioxin response elements (DRE/XRE) in the promoter regions of target genes and upregulate immune checkpoint genes such as VTCN1, thereby facilitating participate in immune escape. Kyn-mediated upregulation of CCL2 and CCL22 further strengthens the immunosuppressive microenvironment, which is consistent with the multiple functions of AhR in regulating inflammation and immune responses.^21^ Importantly, there exists a positive amplification loop between IDO/TDO-mediated kynurenine production and AhR activation. This loop not only enhances the migratory ability of tumor cells, but also regulates the transcription of metabolic enzymes via epigenetic modification including H3K4me3.^19^ Recent research has also demonstrated that AhR can directly modulate IDO2 expression to maintain high kynurenine levels in the tumor microenvironment.^22^ This study clarified the complete positive feedback cascade: Kyn activates AhR to induce EMT and upregulate Snail; Snail overexpression serves as an epigenetic amplifier to boost H3K4me3 enrichment at IDO1/TDO2 promoters to elevate metabolic enzyme expression and kyn synthesis, thus forming a self-amplifying loop. Importantly, loss-of-function SNAI1 silencing cannot reduce basal H3K4me3 modification of IDO1/TDO2 promoters, demonstrating SNAI1 is dispensable for maintaining baseline epigenetic modification. Our ChIP-qPCR data further confirmed that CH-223191 fully abrogates kyn-induced H3K4me3 histone modification at IDO1/TDO2 promoters, directly proving that the epigenetic cascade downstream of kyn strictly depends on AhR activation. Targeting AhR with specific antagonists such as CH-223191 is a promising strategy to block this vicious cycle and reverse immunosuppression.

In vivo experiments using 4T1 orthotopic mouse models verified that elevated kynurenine levels and Snail overexpression accelerate tumor growth in an AhR-dependent manner. As reported previously, kyn, as an endogenous AhR ligand, facilitates malignant phenotypes and immune escape in various tumors.^23^ Snail overexpression also exacerbates tumor progression. The AhR antagonist CH-223191 restored the infiltration and function of CD8⁺ cytotoxic T cells. Consistent with previous reports, CH-223191 can reverse CD8⁺ T-cell exhaustion and immunotherapy resistance induced by the Kyn-AhR axis in lung cancer,^24^ and exert anti-apoptotic effects in chronic lymphocytic leukemia.^25^ These data fully demonstrate the pathological significance of the Kyn-AhR-EMT loop in TNBC and the therapeutic potential of AhR-targeted intervention. Consistent with clinical LC-MS/MS mass spectrometry metabolomics data showing ~16-fold higher intratumoral kynurenine abundance in TNBC compared with normal breast tissue,^18^ our in vivo ELISA quantification of 4T1 orthotopic tumor homogenates verified that exogenous Kyn or Snail overexpression drastically elevates intratumoral kyn levels, and this metabolic shift can be fully reversed by AhR antagonist CH-223191 intervention.

Previous studies on tryptophan metabolism mainly focused on the direct regulation of T-cell differentiation by the Kyn-AhR axis.^26^ For instance, IDO expressed by dendritic cells drives Treg expansion via the IDO-Kyn-AhR axis in atherosclerosis models.^26^ The TDO2-Kyn-AhR pathway modulates tumor dormancy and recurrence in prostate cancer.^27^ Moreover, TDO2 or IDO1 has been proven to promote tumor growth and inhibit T-cell function via the Kyn-AhR pathway in glioma and pancreatic cancer.^28^ ^,^ ^29^ This study first revealed the non-cell-autonomous function of the Kyn-AhR axis inside TNBC cells: activated AhR directly drives EMT and increases the secretion of CCL2/CCL22. Tumor cell-intrinsic changes further recruit immunosuppressive cells and inhibit the function of CD8⁺ T cells to shape an immunosuppressive microenvironment. This finding expands the understanding of the Kyn-AhR axis in tumor progression and suggests the value of targeting intracellular AhR signaling to improve immunotherapy efficacy.

Accumulating evidence has demonstrated the antitumor activity of CH-223191 in various malignancies. In lung adenocarcinoma, CH-223191 restores ferroptosis sensitivity and exerts synergistic antitumor effects with ferroptosis inducers and anti-PD-1 antibodies.^24^ In acute myeloid leukemia, it inhibits tumor cell invasiveness and motility.^30^ In hepatocellular carcinoma, CH-223191 overcomes acquired resistance to lenvatinib and enhances the efficacy of ERK1/2 inhibitor combination therapy.^31^ In chronic lymphocytic leukemia, it blocks the IDO1/Kyn/AhR axis, downregulates MCL1, induces tumor cell apoptosis, and produces synergistic cytotoxicity with venetoclax.^25^ All these findings support the translational potential of CH-223191 as a candidate drug or combination agent for immunotherapy.

This study validated the existence and function of the Kyn-AhR-EMT positive feedback loop based on TNBC cell lines and 4T1 mouse models. Nevertheless, there are several limitations. TNBC exhibits high interpatient heterogeneity, and cell lines and mouse models cannot fully recapitulate the complex microenvironment and genetic background of human TNBC. Further research is required to detect the expression of key molecules in clinical TNBC specimens and analyze their correlations with clinicopathological features and patient prognosis. In addition, only a single AhR antagonist was evaluated in current in vivo experiments. Future studies should test multiple clinical-stage drugs such as IDO1 inhibitors to interrupt this loop, and optimize administration regimens and combination strategies to maximize the efficacy of immunotherapy for TNBC.

## 5. Conclusions

Tryptophan metabolites directly drive EMT in TNBC by activating AhR. Activated AhR further upregulates CCL2/CCL22 to remodel the immunosuppressive microenvironment. In turn, EMT-induced *SNAI1* overexpression epigenetically amplifies the expression of IDO1 and TDO2 via H3K4me3 histone modification, ultimately establishing a positive feedback immune remodeling loop of “tryptophan metabolites-AhR-EMT” (Figure 6). This loop accelerates TNBC invasion and metastasis, induces immunotherapy resistance, and can be used as a prognostic biomarker as well as a promising therapeutic target.

**Figure 6.**
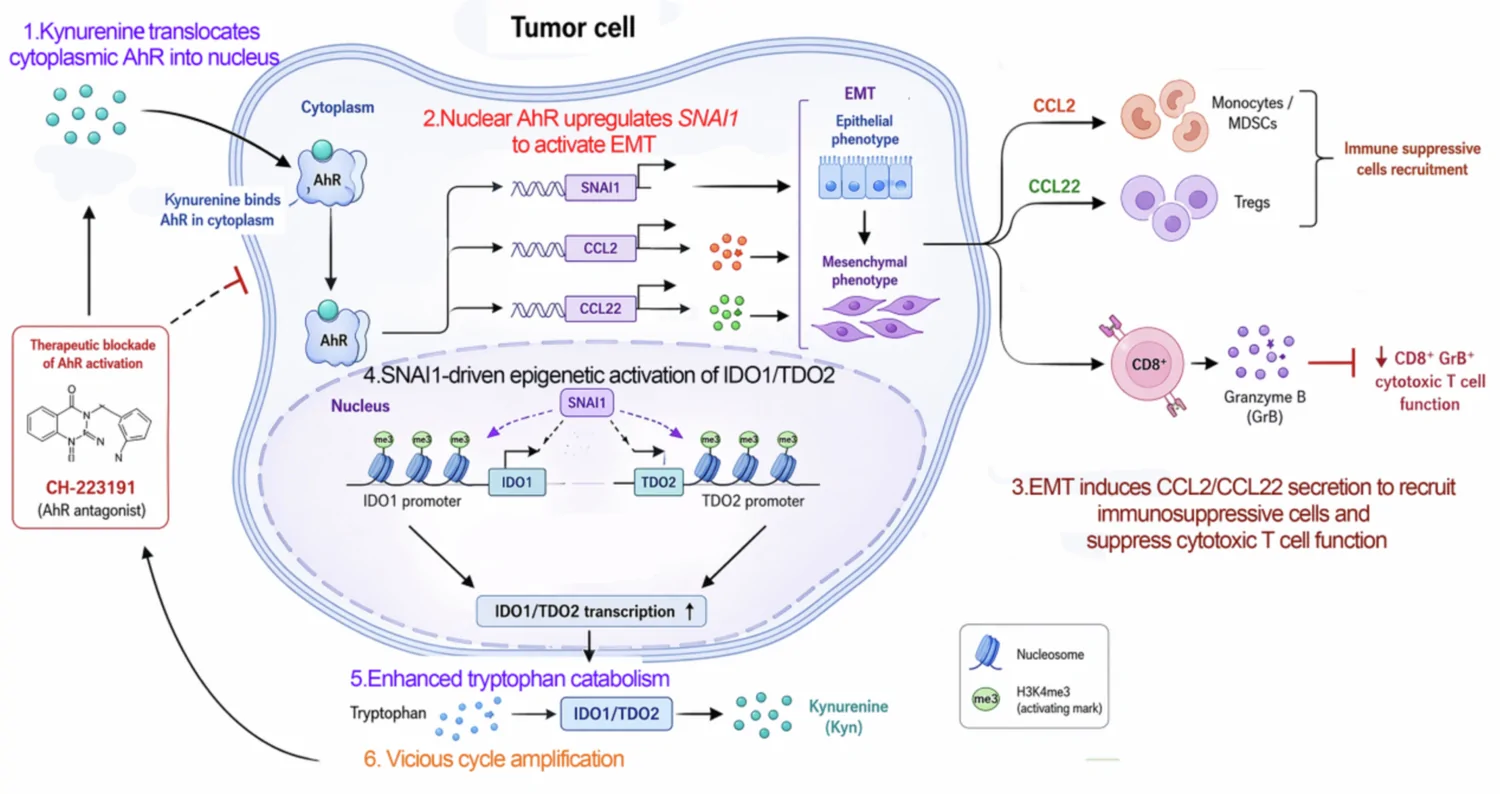
Mechanistic schematic of the Kyn-AhR-EMT positive feedback loop in TNBC. 1. Kynurenine translocates cytoplasmic AhR into the nucleus; 2. Nuclear AhR upregulates *SNAI1* to activate EMT; 3. EMT induces CCL2/CCL22 secretion to recruit immunosuppressive cells and suppress CD8^+^GrB^+^ cytotoxic T-cell function; 4. SNAI1 overexpression drives epigenetic activation of IDO1/TDO2 by enriching the activating histone mark H3K4me3 (SNAI1 is an amplifier rather than an essential maintenance factor for basal H3K4me3 modification); 5. Elevated IDO1/TDO2 accelerates tryptophan degradation to generate more kynurenine; 6. Increased kynurenine levels form a self-amplifying vicious cycle. 7. The AhR antagonist CH-223191 blocks AhR nuclear activation, abolishes kynurenine-triggered H3K4me3 enrichment at IDO1/TDO2 promoters, interrupts the whole feedback loop, and restores antitumor immunity.

## Data Availability

The analyzed data involved in this study are available from the corresponding author upon reasonable request.
